# The Views of a Hospital Secretary

**Published:** 1920-06-19

**Authors:** 


### HOSPITALS AND UNEMPLOYMENT INSURANCE.

### The Views of a Hospital Secretary.

The Unemployment Insurance Bill is, like the National Health Insurance Act, one which affects practically all employed persons. Again, like the Insurance Act, those included are governed by a wage limitation, except in the case of manual workers, who are all insured, irrespective of earnings. In the case of non-manual workers the limitation is £250 per annum. It is suggested that should the Bill become an Act all insured workers will contribute, in the case of males threepence per week, and in the case of females twopence-halfpenny per week, a similar sum being paid by the employer. The method of payment will be on cards by stamps.

Generally speaking, all employed persons insured under the National Health Act will be required to insure against unemployment, but it must be stated that it is possible that some classes of employees, such as nurses and domestic servants, will be allowed to contract out.

The administration of the Act appears to be in the hands of the trade unions and the Labour Exchanges. To obtain benefit under the Act, every employed contributor will, if he or she does not belong to a trade union, have to join a union or arrange to draw the benefit through a Labour Exchange. *There is no alternative.* Those electing to draw the benefit through a trade union will be required to pay an additional sum per week for the increased benefits allowed by the union, whereas those who elected to take benefit through a Labour Exchange will receive only the amount set out in the Bill, *i.e.*, 15s. per week for men and 12s. per week for women. We understand that the benefit for men through a trade union is 20s. per week, and for women 16s. per week.

### Hospital Employees.

The position of hospital employees under the proposed Act is an interesting study not only to those who come within the scope of the Act, but to the committees who are responsible for the management of hospitals. The employee who comes within the Act is faced by one of two alternatives. As soon as the Act becomes operative the majority of insured persons will probably favour the alternative of joining a trade union, and the choice is left entirely to the insured person. The second alternative (Labour Exchange) has distinct disadvantages. If the weekly payment is handed out in the same manner as was the now discontinued unemployment dole, and recipients have to line up outside the Exchange to obtain benefit, the arrangement will prove rather distasteful to many men and women. There remains a trade union, and the question arises, which existing trade union is suitable? Will the mechanics join a skilled trade union and the porters a non-skilled trade union? There remain the clerks, assistant dispensers, laboratory attendants, and many other grades of hospital workers. What is to be their union? If a union is joined the new members will be subject to the rules regarding strikes. A strike, wholly and solely affecting one class of worker is always a grave matter, but when the strike in sympathy with other unions takes place the case becomes one of grave consequence.

Suppose, for example, the "Alpha" trade union declares a strike and it is found in this particular union the majority of non-skilled workers in hospitals are members. Is it to be taken for granted that because of the nature of the work such members will be permitted to "Carry on" as an act of grace by the union leaders? This point is especially important to hospitals while the present industrial unrest continues.

Do managers of hospitals desire to be placed in such a position that, unless they accede to the demand issued by a directorate over which they have no control, and in which they have no voice, the whole of the porters, clerks, or stokers must withdraw their labour or be pronounced "blacklegs"?

The "Omega" union for bank clerks, accountants, stockbrokers' clerks, general clerks, etc., recruiting in their ranks black-coat workers, might be described as a refined and educated type of trade union. Such a union would attract the clerical side of hospital workers. What would be the position generally in hospitals if such a union declared a strike to obtain further concessions for some of their dissatisfied members, or to create a minimum wage irrespective of ability or amount of work performed?

However satisfied might be the hospital worker with his job, he must strike also or his striking fellow-unionist will see to it that his life will be made unendurable by the pickets and other forces of the strike.

What has happened in America? The orderlies employed in the hospitals of New York have organised a local union which has become affiliated with the American Federation of Labour. The local union has at once asked for recognition, employment of union orderlies only, and substantial increases in wages. This action is in itself not greatly to be wondered at, nor necessarily to be deprecated. The sinister feature is the affiliation with a powerful body which has it in its power to stop the supply of the necessaries of life to the inmates of the hospital.

### Why Not a Hospital Trade Union?

There is nothing in the Bill to prevent the formation of a new union. This affords a loophole to hospitals, who can, if they please, form a trade union for their own workers only. It is conceded that even this union will have the power to call a strike on a majority of its members declaring in favour. But there is just this little difference: hospitals are not profit-making and profit-sharing concerns. A large factor in the cause of strikes is the allegations of (quoting from the words of a prominent Labout leader) "the enormous profits

**Hospitals and Unemployment Insurance**—(*continued*).

which are being made by the capitalist through the exploitation of the labouring classes."

It is not for the present writer to offer an opinion on this point, it is sufficiently grateful to him that the hospital worker, at least, cannot share in a grievance shared by other workers.

### Another Factor.

A union of hospital workers, embracing all grades, is, of course, able to strike and to give sympathetic help to another class of worker out for betterment. There is, however, one factor which is worthy of consideration. Any grievance or desire for betterment would be discussed by the committee of a union confined to hospital workers only, and no one outside the Hospital Workers' Union would be allowed to vote. In fact hospital workers would be in the happy position of knowing that only their own fraternity are concerned in the question, whereas if they had no union their concerns would be adjudicated upon by heterogeneous bodies such as the "Alpha" or "Omega" unions, in which but a very small percentage of members belonging to hospitals would or could be included.

The task of forming a union should not be insurmountable. Model rules could be obtained from several unions and adapted or modified according to the requirements of hospital employees.

### The Task Ahead.

The unemployment experienced amongst hospital workers stands very low. Such being the case the benefits could be increased or a reduction made in the weekly contribution necessary to obtain the extra 5s. in the case of men and 4s. in the case of women (the additional weekly sum to be paid to the union for this extra benefit is estimated at 2d., 2½d. or at the very outside 3d. per week).

Whatever is done must be done speedily, for the Act, if passed, becomes operative on October 1 this year.

